# Supplementary material for: Development and validation of a questionnaire, the REST-Q Fire, to identify teamwork-related resources and stressors in firefighting operations
Source: PLoS One. 2024 May 29;19(5):e0304326. doi: 10.1371/journal.pone.0304326 (PMC11135748; doi:10.1371/journal.pone.0304326)
Supplement: S1 Table — (DOCX) [file pone.0304326.s001.docx]

Appendix

**S1 Table. Teamwork-related stressors and resources during firefighting operations**

| **Superordinate categories** | **Subcategories** | **Situation descriptions** | **Items** |
| --- | --- | --- | --- |
| Communication | Passing on information within the squad | One firefighting squad member had important information (e.g., knot in hose; sudden discomfort) to pass on to the other squad member during a firefighting mission. | Information was not passed on at all or too late and/or there was no coordination. (S)  Information was passed on in time and/or brief consultations took place. (R) |
|  | Passing on information between the fire brigade's task forces | During a firefighting operation, a firefighting team member had important information (e.g., situation report; briefing of new squads) to pass on to the other firefighting team members. | Information was not passed on at all or too late and/or there was no coordination. (S)  Information was passed on in time and/or brief consultations took place. (R) |
|  | Quality of information | A firefighting squad member had to relay important information during a firefighting mission. | The information passed on was incorrect, inaccurate, and/or incomplete. (S)  The information passed on was clear, unambiguous, and/or correct. (R) |
|  | Amount of information | Within the fire brigade’s task forces, information was passed on, arrangements were made and the procedure was discussed during a firefighting operation. | There was an unnecessary amount of talking (e.g., too much discussion; too much information at once). (S)  There was as much talking as necessary, but as little as possible (e.g., enough detail but no unnecessary information/discussion). (R) |
|  | Communication with third parties | In a firefighting operation, many people who did not know each other well and do not otherwise work together (e.g., various firefighting units; rail operators/emergency managers; police) had to exchange information and agree on a joint approach. | Information was not passed on at all or was incomprehensible to the other parties involved (e.g., railroad-typical terms; involvement of police and other fire units/firefighting groups). (S)  Information was passed on in a timely and understandable manner (e.g., terms understood by all). (R) |
|  | Shared situational awareness | A firefighter perceived something (e.g., cries for help; hazards at the scene) during a firefighting operation. | The impressions perceived by one firefighter were not passed on to the other firefighters, so that no shared picture of the situation emerged. (S)  The impressions perceived by one firefighter were passed on to the other firefighters and compared with the perceptions of the others, so that a shared picture of the situation was created. (R) |
| Supportive behavior | Watch out for/ check on each other | During firefighting operations at a scene, firefighters from the fire brigade's task forces worked together. | Firefighters did not pay attention to each other (e.g., did not check on each other; did not point out mistakes and/or hazards to others; did not intervene even when hazards were identified) (S)  Firefighters paid attention to each other (e.g., checked on each other; pointed out mistakes and/or (potential) hazards to others; intervened when needed). (R) |
|  | Behavior of team members | During firefighting operations at a scene, firefighters from the fire brigade's task forces worked together. | One or more firefighters behaved in a hectic, selfish, and/or unprofessional manner. This created stress and affected others. (S)  One or more firefighters behaved in a calm and considered, team-oriented manner and/or provided affirmation to other firefighters (e.g., praise; patting on the back). This reduced stress and affected others. (R) |
|  | Respond to the needs of others | A firefighter noted that a member of the fire brigade's task forces needed assistance or encouragement (e.g., sudden discomfort during the operation; experienced psychological distress). | The firefighter ignored the needs of the other and did not provide support. (S)  The firefighter was responsive to the other person's needs and attempted to provide support. (R) |
|  | Supportive behavior/ (support not possible) (2 stressors) | During a firefighting operation at a scene, firefighters from the fire brigade's task forces worked together. | Firefighters did not notice that a member of the group needed assistance and/or did not offer assistance (e.g., one person had to work alone while others had spare capacity; no contribution of ideas/expertise). (S)  Firefighters noticed that a member of the group needed assistance, but were unable to help (e.g., because the path was blocked). (S)  Firefighters noticed that a member of the group needed assistance and assisted when possible (e.g., it was natural for everyone to pitch in when possible; contributing ideas/expertise). (R) |
|  | Reliability of team members | The firefighters of the fire brigade's task forces had their assigned tasks during a firefighting operation and worked at a scene. | Tasks were not performed at all or were not performed conscientiously and/or one or more firefighters did not take their duties seriously. (S)  Tasks were completed reliably and on time and/or the firefighters worked with motivation and the necessary seriousness. (R) |
| Leadership | Hierarchy/ followership | During a firefighting operation, a leader gave an instruction that did not make sense to a firefighter or that could have led to hazardous situations. | The firefighter followed the leader's instructions without voicing his/her concerns or opinion. (S)  The firefighter thought for him/herself and expressed his/her concerns or opinion to the leader. (R) |
|  | Structure/ hierarchy | During a firefighting operation at a scene, firefighters from the fire brigade's task forces worked together with their leaders. | There were no clear leadership structures (e.g., it was not clear who should give which orders to whom) and/or leadership tasks were not performed adequately (e.g., no orders; necessary decisions were not made). (S)  There was a clear structure and hierarchy in the team (e.g., adherence to command structures) and leadership tasks were fulfilled (e.g., maintaining an overview; directing course of action). (R) |
|  | (Insufficient) Consideration of hazards and operational standards | During a firefighting operation at a scene, firefighters from the fire brigade's task forces worked together with their leaders. The leaders had certain tasks (e.g. reconnaissance; hazard assessment), which they should always fulfill. | Potential hazards and/or operational standards were not considered by one or more leaders (e.g., no functioning respirator monitoring; inadequate scene reconnaissance). (S)  One or more leaders considered potential hazards and/or operational standards (e.g., warned of hazards; ensured timely retreat). (R) |
|  | Behavior of leaders | During a firefighting operation at a scene, firefighters from the fire brigade's task forces worked together with their leaders. | One or more leaders behaved in a hectic, uncertain, and/or uncoordinated manner. This created stress and affected others. (S)  One or more leaders behaved in a calm and level-headed, structured and/or supportive manner. This reduced stress and affected others. (R) |
| Shared mental models | Knowledge about common course of action | During a firefighting operation at a scene, firefighters from the fire brigade's task forces worked together to try to achieve the mission objective. | Firefighters lacked information on the course of action and objective of the mission (e.g., sequence of steps; mission strategy). (S)  All firefighters knew the course of action and objective of the mission and everyone knew what to do (e.g., everyone knew the mission tactics and their tasks). (R) |
|  | Knowledge about skills and behavior of team members | During a firefighting operation at a scene, firefighters from the fire brigade's task forces worked together. | The firefighters knew each other poorly or insufficiently (e.g., skills; strengths and weaknesses; personality) and were therefore unsure whether they could rely on each other. (S)  The firefighters knew each other (e.g., skills; strengths and weaknesses; personality) and were therefore able to assess each other well during operations and/or knew whether they could rely on each other. (R) |
|  | Interpositional knowledge | During a firefighting operation at a scene, firefighters from the fire brigade's task forces worked together. Each firefighter had its fixed function and had to exchange information with the other firefighter. | The firefighters did not know enough about the other functions in the firefighting platoon and their duties (e.g., engineer has little knowledge of the water squad's function), so they could not assess what the other person needed at the time (e.g., information) and/or how their own actions affected the others. (S)  The firefighters knew the tasks in the other functions well enough that they could assess what the other person needed (e.g., information) and/or how their own actions affected the others. (R) |
| Organization/ coordination | Task allocation | During a firefighting operation at a scene, many different tasks occurred simultaneously. | Tasks were not distributed at all or not evenly, so some firefighters had to do too many tasks at the same time (e.g., listening in on the radio, issuing materials, and monitoring breathing apparatus). (S)  Tasks were clearly and evenly distributed so that loads were spread and/or no one was overwhelmed with too many tasks. (R) |
|  | Self-initiative and independence (2 stressors) | During a firefighting operation at a scene, firefighters from the fire brigade's task forces worked together. This involved both routine and specialized tasks. | One or more firefighters acted on their own authority during the operation without consultation (e.g., independently changed course of action). (S)  One or more firefighters had to be asked to perform every single step (e.g., put on protective equipment). (S)  The firefighters recognized possible tasks, acted independently, and, if necessary, coordinated work steps on their own initiative (e.g. information about their own course of action). (R) |
|  | Standards and safety measures | During a firefighting operation at a scene, firefighters from the fire brigade's task forces worked together. | Firefighters did not adhere to standards and safety measures (e.g., securing the return path), putting themselves and/or others at risk. (S)  Firefighters adhered to standards and safety measures (e.g., squad stayed together; followed reporting routes), avoiding dangerous situations for themselves and others. (S) |
| Decision-making | (Support of) Decision makers | During a firefighting operation, important decisions had to be made that had an influence on the further course of the operation. | It was not clearly defined who had to make this decision and/or the decision was made by someone who did not have the necessary information/skills. (S)  Decision-making structures were clearly established and/or the decision-maker was provided with the necessary information so that he or she could make the decision. (R) |
|  | Decision in case of situation change/ decide between different alternatives | During a firefighting operation, important decisions had to be made and new information from the environment was constantly coming in. The decisions had an impact on the further course of the operation. | New information about a change in the situation was not used (e.g., new source of fire) to decide on an adjusted course of action. (S)  When an important decision had to be taken, several alternatives were available and firefighters had to decide (e.g., which person would be rescued first; firefighting or human rescue; degree of deviation from accident prevention regulation during human rescue). (S)  Firefighters used new information to reassess the situation and decided to change the plan if necessary. (R) |

Note. S = stressor, R = resource
