# Supplementary material for: Development and validation of a questionnaire, the REST-Q Fire, to identify teamwork-related resources and stressors in firefighting operations
Source: PLoS One. 2024 May 29;19(5):e0304326. doi: 10.1371/journal.pone.0304326 (PMC11135748; doi:10.1371/journal.pone.0304326)
Supplement: Attachment — Submitted filename: Response to reviewers.docx [file pone.0304326.s002.docx]

| Reviewers’ comments | Our responses |
| --- | --- |
| General | |
| R1: Revise the title to reflect objects of the manuscript, Suggestion title: Development and Validation of the REST-Q in firefighting operations. | Thank you for the idea. We integrated validation into the title. Since we find the aspect of non-technical skills essential for this manuscript, we would like to keep this part of the title. |
| R1: REST-Q can be RSTF-Q. | Thank you for your suggestion. We decided for REST-Q since it sounds similar to ‘rescue’ and therefore reflects the content of the questionnaire. |
| R2: The manuscript described a technically sound piece of scientific research about: Development and application of a questionnaire, the REST-Q, to identify teamwork-related resources and stressors in firefighting operations with data that supports the conclusions. Experiments have been conducted rigorously, with appropriate controls, replication, and sample sizes. The conclusions had been drawn appropriately based on the data presented. The research topic , please see attachement. | Thank you for your friendly comment.  Unfortunately, we were not able to find any attachement. It would be great if you could please specify your comment regarding the research topic. |
| R3: This manuscript aims to develop a measurement tool to identify teamwork-related resources and stressors in firefighting operations. The authors interviewed firefighters, then did a document analysis to indicate an overview of the teamwork stressors and resources. Next, they developed the REST-Q questionnaires and conducted them with 491 firefighters. A study like this is important, given the public health implications of the duty-related stresses in fire service personnel. | We appreciate your kind assessment. Thank you. |
| R3: Overall, this is a nicely written paper presenting valuable data on information regarding tools that can be used to identify teamwork-related resources and stressors in firefighting operations. It will significantly contribute to the literature in these critical public health and fire service areas. | Thank you for your friendly comment. We are happy to hear that you value the usefulness of the contribution. |
| Title and Abstract | |
| R1: Abstract conclusion is recommended to include something about: sample mean, standard deviation and Statistical results. | We appreciate your recommendation and included statistical results in the abstract (Page 1, lines 26-32). |
| R3: The title and abstract are appropriate, clear, and informative. Research problems are clear and concise. | Thank you. |
| Introduction | |
| R3: In the introduction section, the authors provided the rationale and the urgency of tool development. | Thank you. |
| Methods | |
| R1: It is best to do an exploratory factor analysis (EFA) first before confirmatory factor analysis (CFA). | We appreciate your thoughtful feedback. This is a point we also discussed in the team and decided to omit the exploratory factor analysis. Since we developed the questionnaire based on our interviews and document analysis, we used the categories identified there. Thus, it was not our intention to examine the data in an exploratory way, but rather to use confirmatory factor analysis to check the structure of the data that we had assumed. Nevertheless, we conducted an exploratory factor analysis, with the result that only one meaningful factor emerged, since the teamwork aspects are closely related. However, from a theoretical point of view, they are distinguishable and can be promoted and trained individually. Therefore, it makes sense for us to still separate them for a practical tool like this. |
| R3: The method section is well-described. The sample size and participants are clearly explained and serve the purpose of this study. However, I would like the authors to clarify participant selection's inclusion and exclusion criteria. For example, in the interview process (Page 6, line 138), the authors stated, "experienced professional, volunteer and industrial firefighters." Please define experienced. Would anyone with work experience higher than three years be included in the study since among those who participated in the interview, their years of work experience varied from 3 to 35 years, regardless of their profession (Page 7, lines 158-167)? Would the gap of years of experience affect their points of view on firefighting operations? Similarly, in the review of the REST-Q, 491 firefighters completed the questionnaire, and they were different in demographics and years of working experience. For example, participants were approximately 95% male and 5% female and had experienced between 1 and 7500 firefighting operations. Would these variables, such as gender or firefighting operations, affect the outcomes of the tools? And this should be included and addressed in the discussion section. | Thank you for your constructive remark. We described the selection criteria more precisely by adding the criteria for experience in firefighting in order to be included in the study (Page 6, lines 137-138).  The further points were included in the limitations where we added more information regarding differences between gender and working experience (Page 22, lines 484-496). |
| R3: The authors stated on Page 11, lines 256-257 that only those participants who completed the respective category in full were used in the CFA. Did 491 participants complete all categories, and were all 491 included in the CFA? Please clarify. | Thank you for your thoughtful question. Not all participants completed all categories. We added the sample size for each category (Page 11, lines 258-263). |
| R3: Also, please specify the N in Tables 2, 3, and 4. | Thank you for the hint. We added the N in Tables 2, 3, and 4. |
| R3: I would also like the authors to explain and define rank frequency and intensity. How do the authors utilize these terms if the rank numbers are different? What is the underly meaning of the mean frequency and mean intensity? They only explained the results from Tables 3 and 4 without further details (Pages 16 and 17). | We appreciate your questions and added further explanation (Page 16, lines 367-374 & page 18, lines 387-393). The ranking lists for frequency and intensity were used separately. We used this approach to find stressors and resources which are at the same time frequent and intense, but we didn’t aggregate those rankings. |
| Discussion | |
| R1: All the participants of this study approximately are male. This need to be included in the limitation and could add recommendation for including more female in the future. | Thank you for your constructive suggestion. We included this remark in the limitations (Page 23, lines 491-496). |
| R3: Some content in the discussion section should move to the result section. Since the study is more into tools development, the authors clearly explained and discussed their findings. | We appreciate your suggestion. We moved some content regarding frequency and intensity of stressors and resources to the results section (Page 16, lines 365-366; Page 18, lines 383-386 & Page 19, lines 398-399). |
