# Supplementary material for: Development and validation of a questionnaire, the REST-Q Fire, to identify teamwork-related resources and stressors in firefighting operations
Source: PLoS One. 2024 May 29;19(5):e0304326. doi: 10.1371/journal.pone.0304326 (PMC11135748; doi:10.1371/journal.pone.0304326)
Supplement: Attachment — Submitted filename: Response to reviewers.docx [file pone.0304326.s003.docx]

Dear reviewers,

Thank you very much for your time and willingness to review our manuscript again and for your valuable comments. We found the comments to be very helpful and have carefully considered and responded to each suggestion. In the following, we will respond to each of your comments in detail and identify the changes in the manuscript. We hope that we have interpreted your comments correctly and that we have handled everything to your satisfaction. All changes in the manuscript are additionally highlighted in yellow to make them easier to follow.

Yours sincerely,

The authors

| Reviewers’ comments | Our responses |
| --- | --- |
| General | |
| R2: The research topic is good and the research problem and objectives have been defined fairly clearly, as the present study contributes to the research on teamwork stressors and resources in firefighting operations by exploring the frequency and intensity of these and relating them to other constructs. | We appreciate your kind assessment. Thank you. |
| R2: The summary of the research was good. However, the researchers have to add the term "questionnaire" to the keywords. | Thank you for the hint. We added “questionnaire” to the keywords. |
| R2: Updating the references in terms of reference to scientific studies and modern scientific references, as there are many old references that the researchers referred to, as there are references for the year 1995. | Thank you for your constructive remark. We updated the references throughout the paper but retained some of the older references as they are also of value. |
| R2: The authors should revise the language to improve readability. | We appreciate your suggestion. The paper was proofread by a native speaker. Nevertheless, we went through the text again very thoroughly. |
| R2: The need to present some important recommendations regarding the development and improvement of questionnaires based on the theoretical framework and field study presented, concerning teamwork stressors and resources in firefighting operations by exploring the frequency and intensity of these and relating them to other constructs, especially with regard to presenting a list of the most important pressures and a list of the required resources that could affect the extinguishing operations. | Thank you for your friendly summary of the paper. The topic is very important for us and we are happy that we were able to present a list of the most important teamwork stressors and resources in firefighting operations. |
| R2: The study has resulted in the REST-Q, a questionnaire that has multiple uses in firefighting education and training evaluation. | Thank you for your friendly comment. We are happy to hear that you value the usefulness of the contribution. |
| R2: The results of the research have scientific and applied importance and value in developing the field of knowledge, despite the lack of reference to scientific studies and modern scientific references, as there are many old references that the researchers referred to, there are references for the year 2002, and many other old references. | Thank you for your constructive remark. We updated the references throughout the paper but retained some of the older references as they are also of value. |
| R2: The lack of theoretical framework for the research to some extent, and the literature related to the subject of the research is accurate, comprehensive, and has been employed accurately. | Thank you. |
| R2: The language used in writing the research report is sound and the phrases are clear. | Thank you for your friendly comment. |
| R2: The tables and figures are correct and clear. However, the references are somewhat outdated and the documentation is according to the journal system, and the method of presenting the research is appropriate according to the scientific rules of writing. | Thank you for your constructive remark. We updated the references throughout the paper but retained some of the older references as they are also of value. For example, Goldstein & Ford (2002) is still relevant as it is a basic literature that elaborates on why it is important to identify specific stressors and resources. |
| R4: Strengthen transition sentences between paragraphs to ensure a seamless flow of ideas. For example, consider using transitional phrases to connect the discussion of environmental stressors to the introduction of teamwork skills. | Thank you for your friendly suggestion. We went through the text thoroughly and included transitional phrases in the suggested and other places. For example, in lines 92-94, we added transitional phrases to connect the description of environmental stressors to the discussion of teamwork skills |
| R4: While FUK is explained upon its first mention, consider providing a brief reminder or explanation if the acronym is used later in the text to aid reader comprehension. | Thank you for the hint. We added an explanation in lines 236-37. |
| R4: Ensure consistent use of terminology throughout the introduction. For instance, check that "teamwork stressors and resources" is consistently referred to by the same terminology. | We checked the consistent use of terminology throughout the whole paper and made adjustments. |
| R4: Depending on the nature of your document, consider incorporating visual aids such as charts or graphs to illustrate the statistical data or complex concepts, enhancing reader understanding. | Thank you for this attractive suggestion. We also believe that a further illustration would be helpful for reading comprehension. We have gladly complied with your request and have created the new figure 1, which serves as an overview of the study procedure. |
| R4: Introduce variety in sentence structure to maintain reader engagement. Mix shorter, impactful sentences with more complex ones for a balanced rhythm. | Thank you for your friendly suggestion. We went through the text thoroughly and tried to create a more balanced rhythm. |
| R4: Be mindful of word choice, opting for the most precise and impactful terms. This can contribute to a more polished and sophisticated writing style. | We appreciate your hint. We went through the text thoroughly and tried to enhance the writing style. |
| R4: While detailed information is important, consider condensing certain sections for better readability. For example, the paragraph at the beginning of Step 2 could be concise. | We appreciate this suggestion. We condensed the paragraph at the beginning of Step 2 and some other sections. |
| R4: Some sentences are lengthy and could be broken down to enhance clarity. Consider breaking down complex sentences into simpler ones for improved clarity. | Thank you for the hint. We went through the text thoroughly and broke down lengthy and complex sentences. |
| Title and Abstract | |
|  | We changed the name of the questionnaire from REST-Q to REST-Q Fire since we found out that there is already a questionnaire named RESTQ. This questionnaire measures the frequency of current stress symptoms along with the frequency of recovery-associated activities in specific versions for athletes (RESTQ-Sport), for coaches (RESTQ-Coach), for adolescents (RESTQ-CA) and for the work context (RESTQ-Work). |
| Introduction | |
| R2: The authors should rewrite their Introduction and Discussion to reference the related literature, especially recently published work. | We appreciate your constructive remark. We added lines 71-91 & 99-102 in the Introduction to reference recently published literature. |
| R4: Elaborate further on the challenges faced by firefighters beyond the statistical data. Providing real-life examples or scenarios can help readers relate more deeply to the issues discussed. | Thank you for the suggestion. We added lines 48-70 which provide two scenarios. |
| Methods | |
| R2: The study needed to determine the validity and reliability of the developed questionnaire. The technical details should be expanded and clarified to ensure that readers understand exactly what the researchers studied. | Thank you for your comment. We weren’t quite sure what the technical details referred to, but we added lines 307-08 to further elaborate on the technical implementation of the questionnaire. |
| R2: One of the positives is the researchers' commitment to the ethics of research work, and this is evident in the research methodology. | Thank you for your friendly comment. |
| R2: The scientific method used in the research is sound. | Thank you. |
| R2: The data and final results are consistent with the research questions and hypotheses. | Thank you. |
| R4: Explicitly mention the sampling strategy used for participant selection. This ensures transparency and helps readers understand the generalizability of your findings. | We appreciate your comment. In order to enhance readability and understanding, we further separated the description into the sections ‘sample’ and ‚procedure‘. Information about the sampling strategy is described in lines 191-95 for the interviews and 281-83 for the online questionnaire. |
| R4: Include information about the response rate in the sample description to provide insights into potential biases. | Thank you for the comment. Unfortunately, we can’t track how many people received the link for the online questionnaire. Thus, we can’t name the response rate. In general, we can deduce that our participants voluntarily completed the questionnaire. |
| R4: Provide more details about the pretesting process, such as the feedback received, and how it informed modifications to the questionnaire. | We appreciate your suggestion and added lines 305-06 about the pretesting process. |
| R4: When discussing the scales used for frequency and intensity, consider providing a brief rationale for choosing these specific scales. | We appreciate your feedback. We added lines 265-69 to explain our choice of scales further. |
| Discussion | |
| R2: The study fails to address how the findings relate to previous research in this area. | Thank you for your friendly hint. We added lines 532-52 to relate the findings to published literature. |
| R2: The authors should rewrite their Introduction and Discussion to reference the related literature, especially recently published work. | Thank you for your friendly hint. We added lines 544-64 to relate the findings to published literature. |
| R2: The results were analyzed, discussed and interpreted accurately and objectively, with specific recommendations linked to the results. | We appreciate your kind assessment. Thank you. |
| R4: While the objectives are stated clearly, briefly clarify how the developed questionnaire will address the identified gaps and contribute to the existing body of knowledge. | Thank you for your suggestion. We added lines 476-81 to clarify how the identified gaps are addressed. |
| R4: Consider grouping related findings together to enhance readability. | Thank you for the comment. We enhanced readability by adding headlines in the discussion section to create a clearer structure. |
| R4: While the strengths are well-highlighted, there could be a more detailed exploration of the limitations, particularly in terms of potential biases and their impact on the study's outcomes.  Consider addressing potential alternative explanations or confounding stressors, resources, and other variables. | We appreciate your suggestion. We added a potential bias regarding the motivation of the participants (lines 583-90).  As already explained in the manuscript, ‘years of experience’ is a confounding variable (lines 592-95). If you have a specific idea in mind for further confounding variables, we are more than willing to discuss that. |
| R4: Expand on the suggestions for future research. Provide more specific recommendations for researchers who might want to build on this study. | We appreciate your constructive remark. We added lines 648-54 & 658-61 which provide more recommendations. |
| R4: Provide additional details on how exactly the REST-Q could be implemented in a training or evaluation setting. Offer examples or scenarios to illustrate its practical use. | Thank you for the suggestion. We added to the description of the implementation in lines 622-28. |
